# Acute Attack Treatment Escalation and Subsequent Relapse Risk in Pediatric Myelin Oligodendrocyte Glycoprotein Antibody-Associated Disease

**DOI:** 10.1212/WNL.0000000000218405

**Published:** 2026-08-07

**Authors:** Silvia Bartolomeo, Riccardo Nistri, Nee Na Kim, Akash Virupakshaiah, Cheryl Hemingway, Olga Ciccarelli, Yael Hacohen

**Affiliations:** 1 Pediatric Neurology, Great Ormond Street Hospital for Children, London, United Kingdom;; 2 Multiple Sclerosis Clinical and Research Unit Department of Systems Medicine, Tor Vergata University, Rome, Italy;; 3 Queen Square MS Centre, Department of Neuroinflammation, UCL Queen Square Institute of Neurology, Faculty of Brain Sciences, University College London, United Kingdom;; 4 Weill Institute for Neurosciences, Department of Neurology, University of California, San Francisco;; 5 NIHR University College London Hospitals Biomedical Research Centre, London, United Kingdom; and; 6 Department of Neuroinflammation, National Hospital for Neurology and Neurosurgery, University College London Hospitals NHS Foundation Trust, United Kingdom.

## Abstract

**Objectives:**

Acute treatment for myelin oligodendrocyte glycoprotein antibody–associated disease (MOGAD) includes high-dose IV methylprednisolone (IVMP), with escalation to IV immunoglobulin (IVIG) and/or plasma exchange (PLEX) based on severity or incomplete response. We evaluated whether escalation at first attack influences disease course.

**Methods:**

We conducted a retrospective single-center study of pediatric-onset MOGAD evaluated between 2015 and 2024. Patients were classified to (1) steroid-only or (2) steroid-plus (IVMP followed by IVIG, PLEX, or both). Outcomes included early (≤1 year) and overall relapses. Time-to-event analyses used Kaplan–Meier and adjusted Cox regression.

**Results:**

Ninety-six of 114 patients were included (58% female; median age 6.3 years [interquartile range (IQR) 4.1–11.3]; median follow-up 3.2 years [IQR 1.4–6.1]). Sixty-eight (71%) received steroid-only therapy and 28 (29%) escalation therapy. Relapses were more frequent with steroid-only treatment at 1 year (28% vs 4%; relative risk [RR] 7.80; 95% CI 1.10–55.3) and at final follow-up (43% vs 11%; RR 3.98; 95% CI 1.33–11.93). Escalation therapy was associated with reduced relapse risk (adjusted hazard ratio [HR] 0.25; 95% CI 0.07–0.85; *p* = 0.026; log-rank *p* = 0.006), consistent across propensity score–adjusted analyses (HR 0.24; 95% CI 0.07–0.81; *p* = 0.022).

**Discussion:**

Acute immunotherapy escalation was associated with lower early and mid-term relapses, suggesting the first attack may represent a therapeutic opportunity to influence disease trajectory.

## Introduction

Myelin oligodendrocyte glycoprotein antibody–associated disease (MOGAD) is a distinct inflammatory demyelinating disorder.^1,2^ Acute treatment includes IV methylprednisolone (IVMP), IV immunoglobulin (IVIG), and/or plasma exchange (PLEX).^3^ In pediatric MOGAD, maintenance therapy is typically only used for relapsing disease.^3^ Emerging adult data suggest early immunotherapy may modify disease trajectory.^4,5^ We investigated whether escalation with IVIG and/or PLEX after IVMP is associated with relapse risk compared with corticosteroids alone.

## Methods

This retrospective, single-center study included patients seen between January 2015 and December 2024. Inclusion criteria were (1) symptom onset before age 18 years, (2) MOGAD diagnosis,^6^ and (3) ≥1 year of follow-up from presentation.

Clinical and demographic data were extracted from electronic medical records, including age at onset, sex, presenting phenotype, acute treatment, and relapse history. Relapse was defined as new or worsening demyelinating symptoms more than 30 days after a prior attack.^6^ Patients were stratified by acute treatment: (1) steroid-only, receiving IVMP at 1 g/d for 3–5 days, followed by an oral prednisone taper over 4–6 weeks. (2) Steroid-plus, receiving IVMP followed by escalation to PLEX (5 cycles), IVIG (2 g/kg), or both. Escalation therapy, defined as the addition of PLEX and/or IVIG after IVMP, was initiated by the treating neurologist based on clinical severity or incomplete early corticosteroid response.

Categorical variables were compared with χ^2^ or Fisher exact tests. Time to first relapse was assessed using Kaplan–Meier estimates, log-rank tests, and adjusted Cox proportional hazards models. To account for treatment assignment within first 30 days, analyses used delayed entry, with patients entering the risk set 30 days after symptom onset. Because escalation therapy was not randomized, multivariable models adjusted for baseline characteristics, within the limitations of a retrospective observational design.

The primary adjusted Cox model included age at onset, sex, and onset phenotype as covariates. The proportional hazards assumption was verified using scaled Schoenfeld residuals. As a sensitivity analysis, a propensity score for receipt of escalation therapy was estimated using the same covariates and entered as a continuous covariate in the Cox model. Secondary sensitivity analyses adjusting for calendar era are reported in the supplementary material.

Analyses were performed using Python, R (v4.4.1), and Stata (version 18; StataCorp, College Station, TX), with statistical significance defined as a two-sided *p*-value <0.05.

### Standard Protocol Approvals, Registrations, and Patient Consents

This study was approved by the Great Ormond Street Hospital Research and Development Department (reference: 16NC10). Given the retrospective design and use of routinely collected clinical data, written informed consent was not required for inclusion.

### Data Availability

Data not published within this article will be made available by reasonable request.

## Results

**Figure 1:**
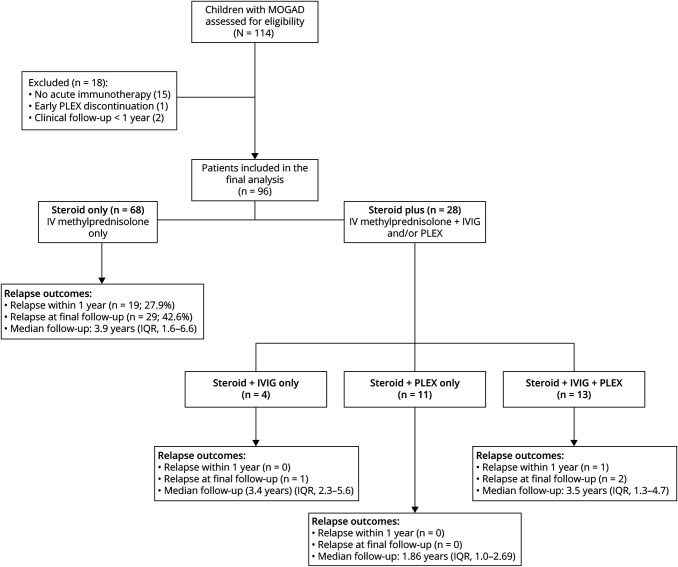
Study Flowchart and Relapse Outcomes Summarizes the study flowchart and reports relapse outcomes in pediatric patients with MOGAD grouped according to acute treatment strategy. IQR = interquartile range; IVIG = IV immunoglobulin; MOGAD = myelin oligodendrocyte glycoprotein antibody–associated disease; PLEX = plasma exchange.

Of 114 patients with MOGAD, 96 met the inclusion criteria and were included in the analysis (Figure 1). Fifty-six (58%) were female; median age at onset was 6.3 years (interquartile range [IQR] 4.1–11.3). Presenting phenotypes were optic neuritis (42/96, 44%), acute disseminated encephalomyelitis (40/96, 42%), cortical encephalitis (9/96, 9%), and myelopathy (5/96, 5%). Sixty-eight (71%) received corticosteroids alone and 28 (29%) received escalation therapy, including PLEX (n = 11), IVIG (n = 4), or both (n = 13); 90 patients (94%) received an oral prednisolone taper.

At a median follow-up of 3.2 years (IQR 1.4–6.1), 32/96 (33%) relapsed; 20/32 (62%) within 1 year. Follow-up duration was shorter in the escalation group. Baseline characteristics were broadly similar, except for a higher frequency of optic neuritis in the steroid-only group (Table 1).

**Table 1:** Demographic and Clinical Characteristics of the Cohort (N = 96)

Variable	Total cohort (n = 96)	Steroid only (n = 68)	Steroid plus (+ IVIG and/or PLEX) (n = 28)	*p* Value
Age at onset, y, median (IQR)	6.33 (4.12–11.33)	6.03 (3.98–9.53)	7.05 (4.88–12.02)	0.222
Female, n (%)	56 (58)	39 (57)	17 (61)	0.823
Onset phenotype, n (%)				
Optic neuritis	42	35 (52)	7 (25)	
ADEM	40	27 (40)	13 (46)	
Cortical encephalitis	9	4 (6)	5 (18)	
Myelopathy	5	2 (3)	3 (11)	
EDSS at nadir, median (IQR)	1.0 (1.0–2.0)	1.0 (1.00–2.0)	1.75 (1.00–3.12)	0.179
Time to steroid, d, median (IQR)	7.0 (3.8–14.2)	7.5 (4.0–16.0)	7.0 (3.0–10.00)	0.256
Time to escalation, d, median (IQR)	9.5 (8.0–15.5)		9.5 (8.0–15.5)	
Oral prednisolone taper, n (%)	90 (94)	64 (94)	26 (93)	>0.99
Follow-up duration, y, median (IQR)	3.17 (1.41–6.12)	3.95 (1.59–6.66)	2.56 (1.27–4.45)	0.062
EDSS at last follow-up, median (IQR)	0.00 (0.00–1.00)	0.00 (0.00–1.00)	0.00 (0.00–1.00)	0.113
Relapse within 1 y, n (%)	20 (21)	19 (28)	1 (4)	0.011
Relapse at final follow-up, n (%)	32 (33)	29 (43)	3 (11)	0.004
Time to first relapse, d, median (IQR)	184 (90–614)	183 (83–561)	1,096 (561–1,278)	0.317

Abbreviations: ADEM = acute disseminated encephalomyelitis; EDSS = expanded disability status scale; IQR = interquartile range; IVIG = IV immunoglobulin; PLEX = plasma exchange.

Values are expressed as median (IQR) or n (%).

Early relapse (within 1 year) occurred in 19/68 (28%) with corticosteroids alone and 1/28 (4%) receiving escalation therapy (relative risk [RR] 7.80; 95% CI 1.10–55.3; *p* = 0.011). At final follow-up, 29/68 (43%) and 3/28 (11%) (RR 3.98; 95% CI 1.33–11.93; *p* = 0.004), respectively relapsed. In the escalation group, 2 relapses occurred after combined PLEX + IVIG (4 months, 3 years) and 1 after IVIG alone (4 years).

**Figure 2:**
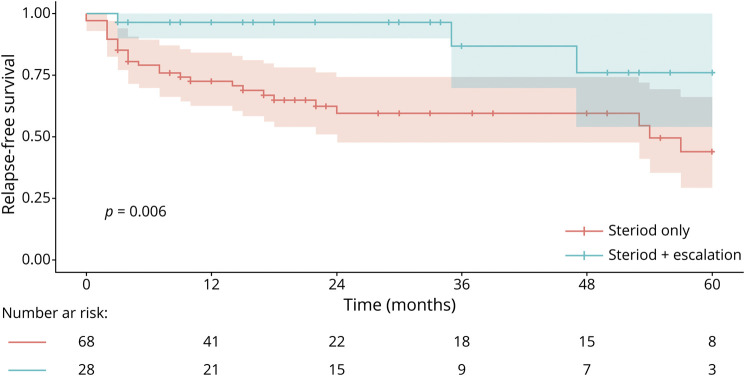
Kaplan–Meier Curves for Time to First Relapse by Acute Treatment Strategy Kaplan–Meier survival curves depict time to first relapse (months) stratified by acute treatment strategy. Patients treated with IV corticosteroids alone experienced earlier relapses compared with those receiving escalation therapy. Separation of curves is most evident early in follow-up. Numbers at risk decrease over time, particularly in the escalation group, reflecting shorter follow-up.

Kaplan–Meier relapse-free survival was significantly higher in the escalation group at 1 year (96.2% vs 71.4%), 2 years (96.2% vs 60.6%), and 5 years (77.7% vs 47.2%; log-rank *p* = 0.006, Figure 2). Multivariable Cox regression confirmed a significantly lower relapse hazard with escalation therapy (hazard ratio [HR] 0.25; 95% CI 0.07–0.85; *p* = 0.026; eTables 1 and 2), consistent with propensity score–adjusted estimates (HR 0.24; 95% CI 0.07–0.81; *p* = 0.022; eTable 3).

## Discussion

In this single-center retrospective cohort, acute immunotherapy escalation with PLEX and/or IVIG after high-dose corticosteroids, at first attack, was associated with substantially lower early and mid-term relapse risk compared with corticosteroids alone, despite similar baseline characteristics and steroid timing, suggesting that acute treatment intensity may shape disease trajectory.

In a multicenter study, early corticosteroids within 7 days, often combined with IVIG, was associated with lower relapse likelihood, whereas delayed therapy increased early and overall relapses.^4^ Further evidence comes from the US Network of Pediatric MS Centers, where nearly half of children with MOGAD relapsed. Female sex and Hispanic/Latino ethnicity predicted higher relapse risk, whereas early maintenance therapy with rituximab or IVIG, but not oral steroids, was protective.^7^ Although focused on preventive therapy, these findings support a role for early intervention. Our results extend this paradigm by showing that escalation during the acute attack itself may lower relapse risk even without long-term maintenance therapy.

Adult MOGAD data also support early acute escalation. In a cohort of 571 attacks, early apheresis (within 4.5 days) improved recovery and disability outcomes compared with delayed use.^8^ Recent international multicenter data support the potential benefit of early PLEX. In 234 patients (42 children), PLEX, mostly combined with corticosteroids, yielded 44% complete recovery and 93% clinical improvement, with delayed PLEX and older age linked to poor recovery.^9^ In a nationwide South Korean cohort, delayed treatment (>5 days) including corticosteroids with or without IVIG or PLEX was associated with a two-fold to three-fold increased relapse risk. Early therapy increased the likelihood of conversion to MOG-IgG seronegativity, suggesting modulation of humoral autoimmunity.^5^

Biologically, these findings are plausible. Inadequately treated acute inflammation may allow persistence and maturation of autoreactive B-cell responses through somatic hypermutation and epitope spreading.^10^ PLEX may rapidly remove circulating antibodies, while IVIG modulates Fc receptor signaling, complement activation, and B-cell function, potentially limiting expansion of pathogenic immune responses, as observed in other antibody-mediated disorders such as AQP4-Ab neuromyelitis optica spectrum disorder.^11^

This study has several limitations. The retrospective, single-center design with nonrandomized, clinician-guided treatment escalation introduces potential confounding by indication. Differences in clinical phenotype, notably higher optic neuritis frequency in the steroid-only group, may have influenced both treatment decisions and relapse risk despite adjustment. Follow-up was shorter and more variable in the escalation group, potentially affecting relapse estimates. A particularly important limitation is the strong collinearity between calendar era and treatment group: 96% of escalation patients had onset in 2018 or later vs 56% in the steroid-only group. Era was independently associated with relapse risk (HR 0.35; *p* = 0.012), reflecting temporal shifts in MOG-IgG test availability, disease recognition, and monitoring intensity that cannot be fully disentangled from the treatment effect. Propensity score, adjusted analyses nonetheless yielded consistent estimates (HR 0.24; *p* = 0.022), supporting but not definitively establishing independence from temporal confounding. These findings require validation in prospective, multicenter cohorts.

None of the patients received maintenance immunotherapy after the first attack, reflecting common practice but potentially limiting applicability to centers that initiate preventive treatment earlier. Residual confounding from unmeasured clinical factors cannot be excluded, and findings should be interpreted as associative rather than causal. Strengths include standardized corticosteroid protocols, extended follow-up, and consistency of findings across multiple analytic approaches, including propensity score adjustment. Prospective multicenter studies are needed to determine whether routine escalation with PLEX and/or IVIG at first MOGAD attack reduces long-term relapse risk and to identify which patients benefit most.
